# Interaction among sea urchins in response to food cues

**DOI:** 10.1038/s41598-021-89471-2

**Published:** 2021-05-11

**Authors:** Jiangnan Sun, Zihe Zhao, Chong Zhao, Yushi Yu, Peng Ding, Jingyun Ding, Mingfang Yang, Xiaomei Chi, Fangyuan Hu, Yaqing Chang

**Affiliations:** Key Laboratory of Mariculture & Stock Enhancement in North China’s Sea, Ministry of Agriculture and Rural Affairs, Dalian Ocean University, Dalian, 116023 China

**Keywords:** Animal behaviour, Marine biology

## Abstract

Interaction among sea urchins remains largely uninvestigated, although the aggregation of sea urchins is common. In the present study, 1, 15 and 30 sea urchins *Strongylocentrotus intermedius* (11.06 ± 0.99 mm in test diameter) were placed in a 1 m^2^ circular tank, respectively. Movement behaviors were recorded for 12 min to investigate potential interactions among sea urchins. After the 12-min control period, we added food cues into the tank and recorded the changes in sea urchins’ behaviors. For the first time, we here quantified the interactions among sea urchins in laboratory and found that the interactions varied with food cues and with different densities. The sea urchins dispersed in random directions after being released. There was no significant difference in the movement speed and the displacement of sea urchins among the three density groups (1, 15 and 30 ind/m^2^). The interaction occurred when sea urchins randomly contacted with the conspecifics and slowed down the movement speed. The speed of sea urchins after physical contacts decreased by an average of 40% in the density of 15 ind/m^2^ and 17% in the density of 30 ind/m^2^. This interaction resulted in significantly higher randomness in the movement direction and lower movement linearity in 15 and 30 ind/m^2^ than in 1 ind/m^2^. After the introduction of food cues, the movement speed, displacement and dispersal distance of sea urchin groups decreased significantly in all the three densities. The dispersal distance and expansion speed of sea urchins were significantly lower in 30 ind/m^2^ than those in 15 ind/m^2^. The present study indicates that the interaction among sea urchins limits the movement of individual sea urchin and provides valuable information into how large groups of sea urchins are stable in places where food is plentiful.

## Introduction

Aggregation of sea urchins is common in nearshore ecosystems^1–4^. Clarifying the grouping behavior involved is important because the aggregation of sea urchins is a precursor to the destructive grazing^5,6^. Group-level patterns are formed by the interactions among the group members^7^. Despite extensive research on sea urchin behaviors^2,8–10^, interaction among sea urchins remains poorly understood. Pearse (1969) described a behavior that disturbed sea urchins *Diadema setosum* and *D. savigny* waved their spines and touched neighbors to wave their spines faster, eventually spreading the stimulus through the group^11^. Physical contact is probably a form of interaction among sea urchins. Field study consistently observed that sea urchins stopped for a few seconds after contacted with conspecific and then changed the movement direction^12^. Aggregation of sea urchins is extensively documented, but there is no solid evidence of the attraction among sea urchins. Most previous studies are not quantitative and are affected by the complexity of the field environments, such as the attraction of food sources. In this study, we quantified the interactions among sea urchins and investigated the effects of interactions on the group behaviors in laboratory.

Food causes significant aggregation of sea urchins^2,13^. Dense urchin grazing frontiers are not only large in scale and widespread in distribution, but long-term persistent^13–17^. Understanding the behavior of sea urchin groups exposed to food cues is thus important to prevent the overgrazing of sea urchins. Sea urchins detect food cues in the water and move toward the source of the cues^2,18^. The mechanism of the stability of the grazing front remains poorly understood^18^. Density is an important characteristic of a group^19,20^. Sea urchin groups of high densities are closely associated with the overgrazing and consequent low-productive barrens^5,21,22^. Field experiment showed that sea urchins aggregated more in a density of 20 ind/m^2^ than of 4 ind/m^2^^23^. The mechanisms by which food and density contribute to the stable aggregation of sea urchin, however, remain mostly unclear.

The sea urchin *Strongylocentrotus intermedius* occur in intertidal and shallow waters of China, Japan and Korea^24^. Small *S. intermedius* (10–15 mm) are also reseeded for stock enhancement in both China and Japan^25^. Full understanding on the interaction among small *S. intermedius* in response to food cues has important implications for the management of local nearshore ecosystems. We here recorded the behavior of 1, 15 and 30 small *S. intermedius* (11.06 ± 0.99 mm in test diameter) in a 1 m^2^ circular tank for 12 min after the release of food cues into the tank. Based on our previous records of speed of movement of small *S. intermedius* (0.34 ± 0.10 mm/s)^26^, 1 m^2^ circular tank and 12-min experiment period allow sea urchins to move freely and allow us to observe possible interactions among sea urchins. Before food cues experiments, movement behavior of small *S. intermedius* was recorded for 12 min as the control. The main purposes of the present study are to investigate: (1) whether sea urchins interact with conspecifics; (2) whether interactions affect the behaviors of sea urchins at different densities; (3) whether food cues affect the interaction among sea urchins at different densities.

## Results

### Individual behaviors and interactions

**Figure 1:**
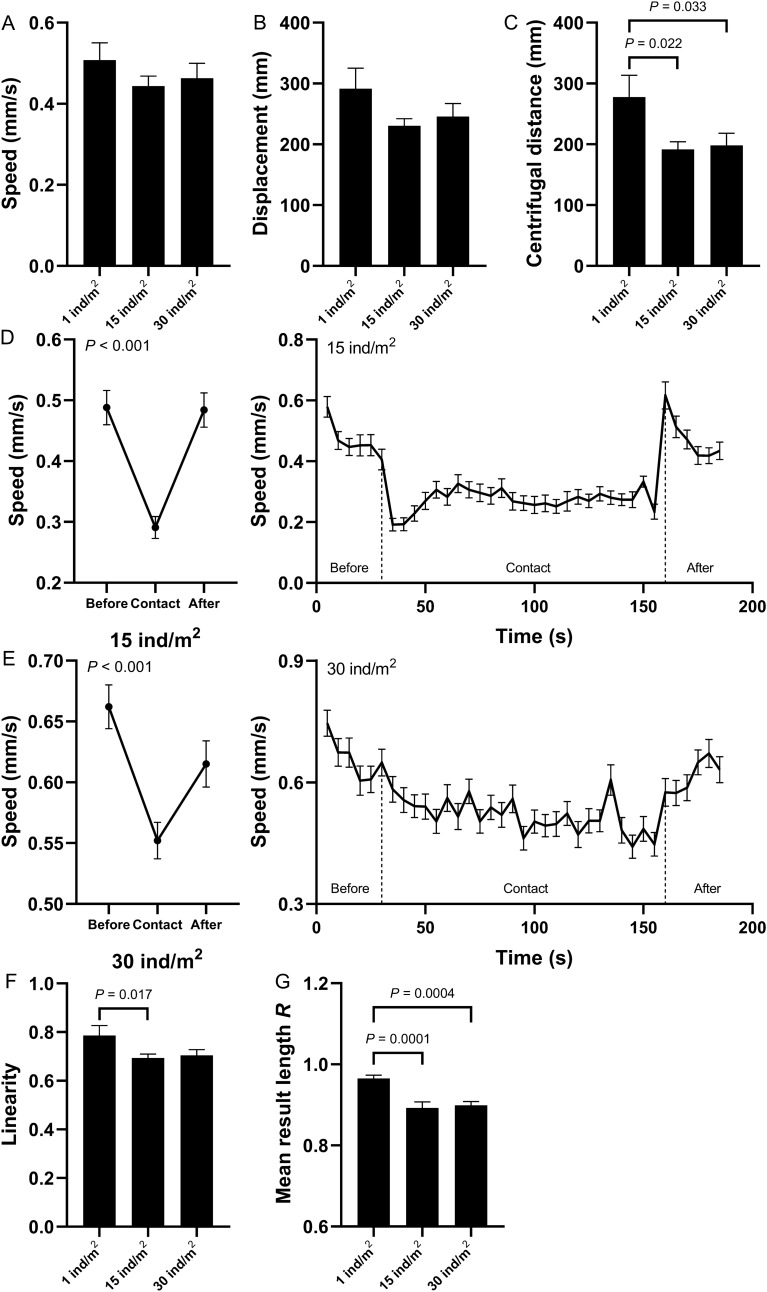
Behavioral changes (mean ± SEM) of sea urchins when they randomly contacted conspecifics in different density groups without the food cues. Movement speed, displacement and centrifugal distance of sea urchins in the 1, 15 and 30 ind/m^2^ groups without the food cues (**A**–**C**). Speed of sea urchins before, during and after the contacts in 15 ind/m^2^ (**D**, left) and the speed per 5 s (**D**, right). Speed of sea urchins before, during and after contact in 30 ind/m^2^ (**E**, left) and the speed per five seconds (**E**, right). Movement linearity and mean result length *R* of sea urchins in 1, 15 and 30 ind/m^2^ without the food cues (**F**,**G**).

There was no significant difference in the movement speed and displacement during the 12 min of observations among the three densities of sea urchins (0.51 ± 0.04 mm/s and 291.60 ± 33.53 mm in 1 ind/m^2^; 0.44 ± 0.02 mm/s and 230.45 ± 11.67 mm in 15 ind/m^2^; 0.46 ± 0.04 mm/s and 245.72 ± 21.40 mm in 30 ind/m^2^, Fig. 1A,B; Supplementary Tables S1 and 2). By calculating the distance between the sea urchin and the center of the tank after 12 min ($CD = \sum \sqrt {\left[ {x_{i} - x_{tank} } \right]^{2} + \left[ {y_{i} - y_{tank} } \right]^{2} } \times k/n$, (*x*_*i*_*, y*_*i*_) is the coordinate of sea urchin *i* at the end of the period, (*x*_*tank*_*, y*_*tank*_) is the center coordinates of the tank,* k* is the scale of the picture, *n* is the number of sea urchins in different density group), the mean centrifugal distance was significantly different among the three density groups. Individual sea urchins (1 ind/m^2^) moved significantly farther outward from the center than those in 15 and 30 ind/m^2^ (277.84 ± 35.70 mm in 1 ind/m^2^; 191.40 ± 12.65 mm in 15 ind/m^2^; 198.26 ± 19.92 mm in 30 ind/m^2^, Fig. 1C; Supplementary Tables S1 and 2). Interaction occurred when sea urchins randomly contacted conspecifics (total numbers: 67 in 15 ind/m^2^, 203 in 30 ind/m^2^; average time: 120.37 ± 13.75 s in 15 ind/m^2^, 121.43 ± 8.30 s in 30 ind/m^2^). The physical contact significantly slowed down the movement speed (Fig. 1D,E; Supplementary Table S3). By comparing the distance and displacement of the sea urchins, we obtained the linearity *l* of free movement of sea urchins in 12 min ($l_{i} = \frac{{displacement_{i} }}{{distance_{i} }}$. *Displacement*_*i*_ is the movement displacement of sea urchin *i* after 12 min. *Distance*_*i*_ is the total movement length of sea urchin *i* within 12 min). The motion linearity of sea urchins in 15 ind/m^2^ was significantly lower than that of individual sea urchin in 1 ind/m^2^ (0.79 ± 0.04 in 1 ind/m^2^; 0.69 ± 0.02 in 15 ind/m^2^; 0.70 ± 0.02 in 30 ind/m^2^, Fig. 1F; Supplementary Tables S1 and 2). *R* is a measure of the randomness of movement, where 0 is completely random movement and 1 is completely directional movement ($R = \sqrt {\left[ {\frac{1}{n}\sum\nolimits_{t = 1}^{n} {\cos } \left( {\theta_{t} } \right)} \right]^{2} + \left[ {\frac{1}{n}\sum\nolimits_{t = 1}^{n} {\sin } \left( {\theta_{t} } \right)} \right]^{2} }$, *θ*_*t*_ represents the turn angle at *t* minutes, *n* = 12 min). The randomness of sea urchin movement in groups (15 and 30 ind/m^2^) was significantly higher than that of sea urchins in 1 ind/m^2^ (0.97 ± 0.01 in 1 ind/m^2^; 0.89 ± 0.01 in 15 ind/m^2^; 0.90 ± 0.01 in 30 ind/m^2^, Fig. 1G; Supplementary Tables S1 and 2).

### Food cue

**Figure 2:**
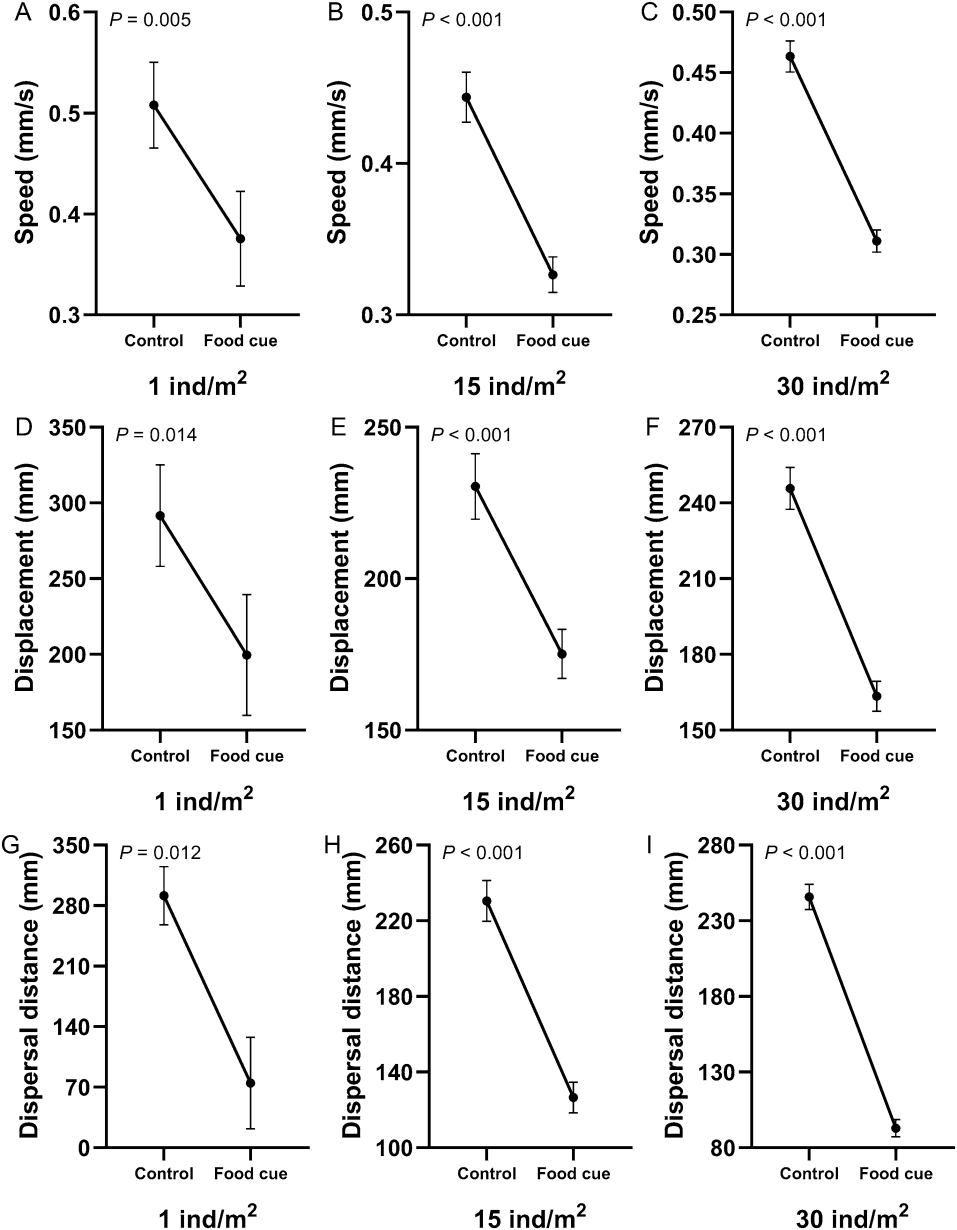
Behavioral changes (mean ± SEM) of individuals in response to the food cues. Movement speed of sea urchins without and with the food cues in the three density groups (**A**–**C**). Movement displacement of sea urchins with and without the food cues in the three density groups (**D**–**F**). Dispersal distance of sea urchins with and without the food cues in the three density groups (**G**–**I**).

**Figure 3:**
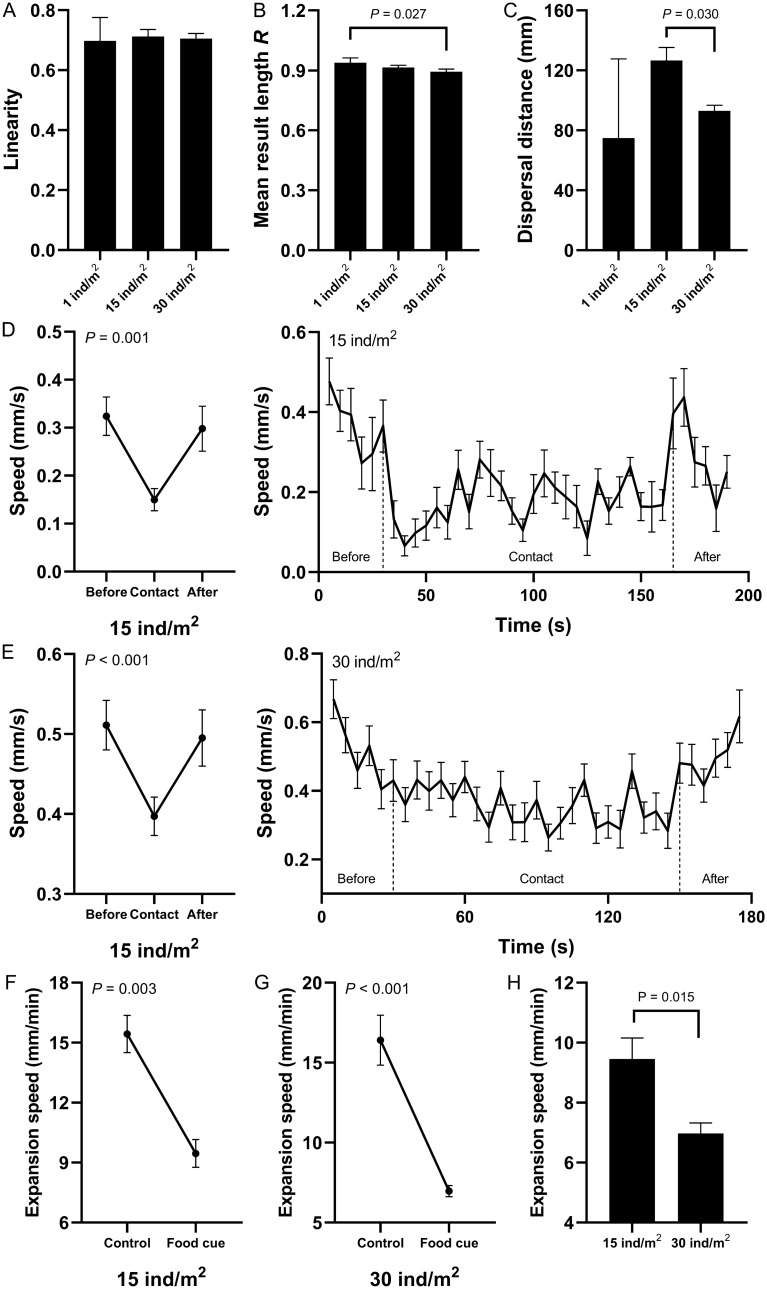
Behavioral changes (mean ± SEM) of sea urchins exposed to the food cues when they randomly contacted conspecifics in different density groups. Movement linearity, mean result length *R* and dispersal distance of sea urchins exposed to the food cues in 1, 15 and 30 ind/m^2^ (**A**–**C**). Speed of sea urchins before, during and after the contact in 15 ind/m^2^ when exposed to the food cues (**D**, left) and speed per 5 s (**D**, right). Speed of sea urchins before, during and after the contact in 30 ind/m^2^ when exposed to the food cues (**E**, left) and speed per 5 s (**E**, right). Expansion speed of sea urchins exposed to the food cues in 15 and 30 ind/m^2^ (**F**–**H**).

Food cues (the kelp *Saccharina japonica*) were added to the tank after 12 min of free activity (the control period) in the three density groups. After the release of the food cues, the movement speed and displacement of sea urchins decreased significantly in all the three density groups (Fig. 2A–F; Supplementary Table S1 and 4). Dispersal distance *r* is the movement distance of each sea urchin from its initial position ($r = \sum (\sqrt {\left[ {x_{i} - x_{0} } \right]^{2} + \left[ {y_{i} - y_{0} } \right]^{2} } - \sqrt {\left[ {x_{p} - x_{0} } \right]^{2} + \left[ {y_{p} - y_{0} } \right]^{2} } ) \times k/n$, (*x*_*0*_*, y*_*0*_) is the coordinates at the beginning of the experiment, (*x*_*p*_*, y*_*p*_) is the initial position of each period, *n* is the number of sea urchins in different density group). In the food cues period, dispersal distance of sea urchins significantly lower than that in the control period in the three density groups (74.91 ± 52.81 mm in 1 ind/m^2^; 126.57 ± 8.72 mm in 15 ind/m^2^; 92.96 ± 3.81 mm in 30 ind/m^2^, Fig. 2G–I; Supplementary Tables S1 and 4). There was no significant difference in motion linearity among the three groups (Fig. 3A; Supplementary Table S1 and 2). The motion randomness of sea urchin in 30 ind/m^2^ was significantly higher than that sea urchins in 1 ind/m^2^ (0.94 ± 0.02 in 1 ind/m^2^; 0.91 ± 0.01 in 15 ind/m^2^; 0.89 ± 0.01 in 30 ind/m^2^, Fig. 3B; Supplementary Tables S1 and 2). The dispersal distance of sea urchins in 30 ind/m^2^ was significantly lower than that of sea urchins in 15 ind/m^2^ (Fig. 3C; Supplementary Table S2). Sixteen contacts occurred in 15 ind/m^2^ (an average of 122.50 ± 25.85 s) and 64 in 30 ind/m^2^ (an average of 107.81 ± 11.45 s). The physical contact significantly decreased the speed of sea urchins (Fig. 3D,E; Supplementary Table S3). Expansion speed *v*_*e*_ (mm/min) is the average speed of all sea urchins in the group from the group center ($v_{e} = \sum \left( {\sqrt {\left[ {x_{i} - x_{c} } \right]^{2} + \left[ {y_{i} - y_{c} } \right]^{2} } - \sqrt {\left[ {x_{p} - x_{c0} } \right]^{2} + \left[ {y_{p} - y_{c0} } \right]^{2} } } \right) \times \frac{k}{12}/n$, (*x*_*c*_*, y*_*c*_)/(*x*_*c0*_*, y*_*c0*_) is the mean of all the sea urchin coordinates in the group at the end/beginning of each period, *n* is the number of sea urchins in different density group). The expansion speed of sea urchins in 15 and 30 ind/m^2^ decreased significantly in response to the food cues and showed significant difference between the two groups (15.44 ± 0.93 mm/min and 9.46 ± 0.70 mm/min in 15 ind/m^2^; 16.41 ± 1.56 mm/min and 6.71 ± 0.35 mm/min in 30 ind/m^2^, Fig. 3F–H; Supplementary Table S5).

## Discussion

In this study, interaction occurred when sea urchins randomly contacted conspecifics. This physical contact had negative effects on the movement of sea urchins, in which the speed of sea urchins dropped by about 40% in 15 ind/m^2^ and 17% in 30 ind/m^2^. This explains the field phenomenon that sea urchins stop for a few seconds after contact with a conspecific^12^. Due to the interaction among sea urchins, the motion linearity of sea urchins in groups was significantly lower than that of individual sea urchins, while the randomness of movement direction was significantly higher. After being released, the sea urchins in the group diverged in all directions. Moving away from each other would reduce the negative effect of interaction. This is consistent with the field observation that the dispersal among sea urchins reduces the competition within sea urchin groups^27^. Stock enhancement is an important approach to the increasing marketing requires in both China and Japan^25^. In China, for example, ~ 30% of artificially bred *S. intermedius* seeds are released into the sea for the local stocking (e.g. 2.7 million in 2017)^25^. Rapid dispersal of *S. intermedius* is important to prevent the formation of high-density aggregations and the negative effects of increased stocking density on small *S. intermedius*^28,29^. The present study found that the high-density group formed at the beginning prevented the dispersion of sea urchins within the group. The explanation is that interactions within the group negatively affect the movement behaviors of sea urchin (e.g. movement speed). Thus, we suggest that high density is not appropriate during the reseeding of *S. intermedius* because of the negative interactions among sea urchins.

After the release of the food cues, movement speed of sea urchins significantly slowed down in all the three density groups. This behavioral strategy increases the duration of sea urchins in food patches^8^. In this experiment, sea urchins did not form significant aggregations under the condition of evenly distributed food. This is different from the results of food-induced sea urchin aggregation reported in the field^2,13,30^. This difference indicates that the aggregation of sea urchins highly depends on condition of the food sources. The negative effects of sea urchin interactions on movement consistently existed with the exposure to food cues. Further, we found that dense sea urchin groups spread out significantly less. The low mobility of sea urchins was thought to be a factor in the stable existence of a dense sea urchin on grazing front^12^. The present study suggests that low mobility of a grazing front is not only due to the decrease in sea urchins’ speed in response to food cues, but the constrained expansion caused by the interaction among the sea urchins.

The present study quantified the interaction among sea urchins and found negative impacts of sea urchins on the movement of conspecifics. This effect slowed the outward expansion of the sea urchin group and increased with the increasing density. Food cues caused sea urchins to slow down. Large groups spread more slowly in response to food cues due to the interaction among sea urchins. The present study suggests that high density is not appropriate in reseeding because of the adverse interaction among sea urchins.

## Methods

### Sea urchins

368 small *S. intermedius* (11.06 ± 0.99 mm in test diameter, 6.02 ± 0.66 mm in test height and 0.76 ± 0.21 g in body weight) used in the experiment were transported to the Key Laboratory of Mariculture & Stock Enhancement in the North China’s Sea, Ministry of Agriculture and Rural Affairs, Dalian Ocean University (121° 37ʹ E 38° 87ʹ N) in December 2019. Before the experiments, the sea urchins were maintained at 12 ± 0.5 °C in a 300 L tank in the laboratory and were fed ad libitum fresh brown algae *Gracilaria lemaneiformis*. All applicable international, national, and/or institutional guidelines for the care and use of animals were followed by us.

### Interactions among sea urchins

**Figure 4:**
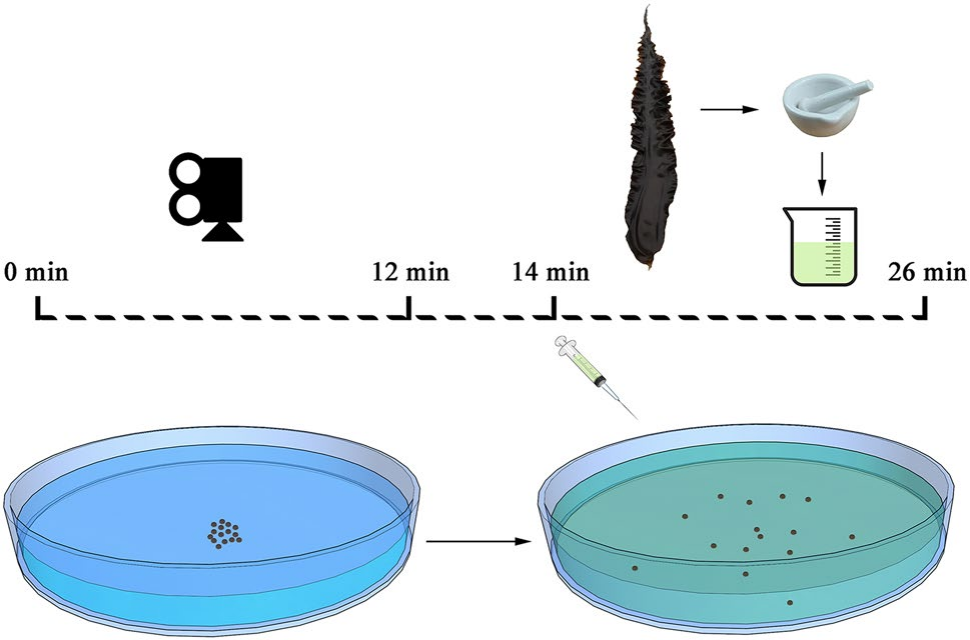
Diagram of the experiment. The period without the food cues was from 0 to 12 min, the period with the food cues was from 14 to 26 min.

To investigate whether sea urchins interact with conspecifics, we measured the behaviors of 1, 15 and 30 sea urchins in a 1 m^2^ circular tank, respectively. Fresh seawater was filled into the experimental device in a depth of 3 cm at the same temperature as the sea urchin’s holding tank (12 ± 0.5 °C). Sea urchins were gently placed in the center of the device. There was no contact among sea urchins in groups at the beginning of the experiment (Fig. 4). The behaviors of individuals in the three groups were recorded for 12 min using a video camera (Legria HF20; Canon, Tokyo, Japan) without external food cues. Experiments were repeated eight times using different sea urchins for all the three densities (n = 8).

### Behaviors and interactions among sea urchins exposed to food cue

After the 12 min of observation, we added 100 mL of the food cues over a period of two minutes and then recorded the response of the sea urchins to the food cues for another 12 min. The whole experiment lasted 26 min (Fig. 4). In the experiment, the food cues were produced by adding 20 g dry powder of kelp (*S. japonica*) to 100 mL fresh seawater and then filtering it three times with a bolting-silk net (44 µm in mesh size). The experiment was conducted at a low light intensity (8 lx). We repeated the experiment eight times using different sea urchins and changed the seawater for all three densities (n = 8).

### Behaviors

Images from the 26-min video were taken every 5 s. A total of 313 images were combined into a stack using the software ImageJ (1.52 s version). We designated two periods of images in the stack: the period without food cues from 1 to 145 images, the food cue period from 169 to 313 images. For each video, we used the plugin manual tracking in ImageJ (1.52 s version) to extract the coordinates of each sea urchin.

We calculated the movement speed (*v*), and displacement (*d*) of the sea urchins as follows:

1 $$ \begin{array}{*{20}c} {v_{i} = \sqrt {\left[ {x_{i} \left( t \right) - x_{i} \left( {t - 1} \right)} \right]^{2} + \left[ {y_{i} \left( t \right) - y_{i} \left( {t - 1} \right)} \right]^{2} } \times \frac{k}{5}} \\ \end{array} , $$

2 $$ \begin{array}{*{20}c} {d_{i} = \sqrt {\left[ {x_{i} \left( t \right) - x_{p} } \right]^{2} + \left[ {y_{i} \left( t \right) - y_{p} } \right]^{2} } \times k} \\ \end{array} , $$

where (*x*_*i*_(*t*)*, y*_*i*_(*t*)) is the coordinates of sea urchin *i* at image *t*, (*x*_*p*_*, y*_*p*_) is the initial position of each period of the sea urchin,* k* is the scale of the picture.

During the experiment, sea urchins were considered in contact when they had physical contact with a conspecific (the touch of spines). Movement speeds were recorded from the beginning of contact to the separation of the sea urchins (no physical contact). The movement speeds in the 30 s before and after the contact process were recorded as the behavior before and after contact.

To measure the random level of movement, we measured the mean result length *R* of movement per minute for 12 min. A value of 0 indicates absolute random motion and a value of 1 indicates absolute directional movement^31^.

3 $$ \begin{array}{*{20}c} {R = \sqrt {\left[ {\frac{1}{n}\mathop \sum \limits_{t = 1}^{n} \cos \left( {\theta_{t} } \right)} \right]^{2} + \left[ {\frac{1}{n}\mathop \sum \limits_{t = 1}^{n} \sin \left( {\theta_{t} } \right)} \right]^{2} } } \\ \end{array} , $$

where *θ*_*t*_ represents the turn angle at time *t*.

We measured the linearity *l* in each period using the ratio of displacement (*d*) to distance after 12 min^32^.

4 $$ \begin{array}{*{20}c} {l_{i} = \frac{{displacement_{i} }}{{distance_{i} }}} \\ \end{array} . $$

We calculated the distance of the sea urchins from their initial position at the end of the experiment. We used the average of this distance within the sea urchin group to indicate the spread *r* of different density groups.

5 $$ \begin{array}{*{20}c} {r = \sum \left( {\sqrt {\left[ {x_{i} - x_{0} } \right]^{2} + \left[ {y_{i} - y_{0} } \right]^{2} } - \sqrt {\left[ {x_{p} - x_{0} } \right]^{2} + \left[ {y_{p} - y_{0} } \right]^{2} } } \right) \times k/n} \\ \end{array} , $$

where (*x*_*i*_*, y*_*i*_) is the coordinate of sea urchin *i* at the end of the period, (*x*_*p*_*, y*_*p*_) is the initial position of each period, (*x*_*0*_*, y*_*0*_) is the coordinate at the beginning of the whole experiment, *k* is the scale of the picture, *n* is the number of sea urchins in different density group (15 ind/m^2^ group: *n* = 15, 30 ind/m^2^ group: *n* = 30).

The expansion speed of the sea urchin groups was compared with the distance of the individual from the center of the group. The center coordinates of the group (*x*_*c*_*, y*_*c*_) are the mean of all the sea urchin coordinates in the group at the end of each period. Expansion speed *v*_*e*_ (mm/min) is the average speed from group center for all sea urchins in each group.

6 $$ \begin{array}{*{20}c} {v_{e} = \sum \left( {\sqrt {\left[ {x_{i} - x_{c} } \right]^{2} + \left[ {y_{i} - y_{c} } \right]^{2} } - \sqrt {\left[ {x_{p} - x_{c0} } \right]^{2} + \left[ {y_{p} - y_{c0} } \right]^{2} } } \right) \times \frac{k}{12}/n} \\ \end{array} , $$

where (*x*_*i*_*, y*_*i*_) is the coordinate of sea urchin *i* at the end of the period, (*x*_*p*_*, y*_*p*_) is the initial position of each period, (*x*_*c*_*, y*_*c*_) / (*x*_*c0*_*, y*_*c0*_) is the mean of all the sea urchin coordinates in the group at the end/beginning of each period.

### Statistical analysis

The data were tested for homogeneity of variance and normal distribution before all statistical analyses using the Levene test and Kolmogorov–Smirnov test, respectively.

In the period without food cues, movement speed, displacement, centrifugal distance, linearity and mean result length *R* of sea urchins among the three groups were analyzed using one-way ANOVA (1 ind/m^2^ group: n = 8; 15 ind/m^2^ group: n = 8; 30 ind/m^2^ group: n = 8). Mann–Whitney U test was used if the data did not meet the normal distribution and/or variance. One-way repeated measures ANOVA was used to compare movement speeds of the sea urchin before, during, and after contact.

In the food cues experiments, paired sample T test was used to compare the movement speeds, displacement and expansion speed of sea urchins in response to the food cues (1 ind/m^2^ group: n = 1 × 8 = 8; 15 ind/m^2^ group: n = 15 × 8 = 120; 30 ind/m^2^ group: n = 30 × 8 = 240). Wilcox signed-rank test was used for the data that did not satisfy the normal distribution. Movement linearity, mean result length *R* and dispersal distance among the three groups were analyzed using One-way ANOVA. Mann–Whitney U test was used when the data did not meet the normal distribution and/or variance. The independent sample T test was used to compare the speed and expansion speed between 15 and 30 ind/m^2^ group after the food cues was released.

All data analyses were performed using SPSS 25.0 statistical software. A probability level of *P* < 0.05 was considered as significant.

### Ethical approval

All applicable international, national, and/or institutional guidelines for the care and use of animals were followed by the authors.

## Supplementary Information

Supplementary Information 1.

Supplementary Information 2.

Supplementary Video 1.

Supplementary Video 2.

Supplementary Video 3.

## Data Availability

All data generated or analyzed during this study are included in this published article (and its Supplementary Information files).
