# Supplementary material for: Interaction among sea urchins in response to food cues
Source: Sci Rep. 2021 May 11;11:9985. doi: 10.1038/s41598-021-89471-2 (PMC8113249; doi:10.1038/s41598-021-89471-2)
Supplement: Supplementary file 2 — Supplementary Information 2. [file 41598_2021_89471_MOESM2_ESM.docx]

**Supplementary information for**

**Interaction among sea urchins in response to food cues**

Jiangnan Sun, Zihe Zhao, Chong Zhao*, Yushi Yu, Peng Ding, Jingyun Ding, Mingfang Yang, Xiaomei Chi, Fangyuan Hu, Yaqing Chang*

Key Laboratory of Mariculture & Stock Enhancement in North China's Sea, Ministry of Agriculture and Rural Affairs, Dalian Ocean University, Dalian 116023, China.

*email: chongzhao@dlou.edu.cn (C Zhao), changlab@hotmail.com (Y Chang)

**Supplementary Table 1**

**Data of individual behaviors (mean ± SEM).** Data of movement speed (mm/s), displacement (mm), mean result length *R*, motion linearity, centrifugation distance *CD* (mm) and dispersal distance *r* (mm) of sea urchins in 1 ind/m^2^, 15 ind/m^2^ and 30 ind/m^2^ in the control and food cues period.

|  | | 1 ind/m^2^ | | 15 ind/m^2^ | | 30 ind/m^2^ | |
| --- | --- | --- | --- | --- | --- | --- | --- |
|  | | control | food cue | control | food cue | control | food cue |
| Speed | 0.51 ± 0.04 | | 0.38 ± 0.05 | 0.44 ± 0.02 | 0.33 ± 0.02 | 0.46 ± 0.04 | 0.31 ± 0.02 |
| Displacement | 291.60 ± 33.53 | | 199.61 ± 39.82 | 230.45 ± 11.67 | 175.10 ± 8.53 | 245.72 ± 21.40 | 163.42 ± 9.25 |
| *R* | 0.97 ± 0.01 | | 0.94 ± 0.02 | 0.89 ± 0.01 | 0.91 ± 0.01 | 0.90 ± 0.01 | 0.89 ± 0.01 |
| Linearity | 0.79 ± 0.04 | | 0.70 ± 0.08 | 0.69 ± 0.02 | 0.71 ± 0.02 | 0.70 ± 0.02 | 0.71 ± 0.02 |
| *CD* | 277.84 ± 35.70 | | 369.28 ± 40.61 | 191.40 ± 12.65 | 330.88 ± 13.28 | 198.26 ± 19.92 | 308.10 ± 24.71 |
| *r* | 291.60 ± 33.53 | | 74.91 ± 52.81 | 230.45 ± 11.67 | 126.57 ± 8.72 | 245.72 ± 21.40 | 92.96 ± 3.81 |

**Supplementary Table 2**

***P* values of analysis on behaviors among the three density groups.** One-way ANOVA was used to compare the movement speed (mm/s), displacement (mm), mean result length *R*, motion linearity and centrifugation distance *CD* (mm) of sea urchins among 1 ind/m^2^, 15 ind/m^2^ and 30 ind/m^2^. Mann-Whitney U test was used if the data did not meet the normal distribution and/or variance.

|  |  | Speed | Displacement | *R* | Linearity | *CD* | *r* |
| --- | --- | --- | --- | --- | --- | --- | --- |
| Control | 1-15 | 0.213 | 0.085 | < 0.001 | 0.017 | 0.022 | 0.085 |
|  | 1-30 | 0.383 | 0.190 | < 0.001 | 0.078 | 0.033 | 0.190 |
|  | 15-30 | 0.698 | 0.657 | 0.673 | > 0.900 | 0.846 | 0.687 |
| Food cue | 1-15 | 0.276 | 0.480 | 0.198 | 0.829 | 0.352 | > 0.900 |
|  | 1-30 | 0.155 | 0.301 | 0.027 | 0.911 | 0.144 | 0.183 |
|  | 15-30 | 0.724 | 0.735 | > 0.900 | 0.917 | 0.577 | 0.030 |

**Supplementary Table 3**

**Movement speeds (mm/s) of sea urchins before, during and after the physical contact in 15 ind/m^2^ and 30 ind/m^2^ (mean ± SEM).** One-way repeated measures ANOVA was used to compare speeds of sea urchins before, during and after physical contact in 15 ind/m^2^ and 30 ind/m^2^, respectively.

|  |  | Before | Contact | After | *P* |
| --- | --- | --- | --- | --- | --- |
| Control | 15 | 0.49 ± 0.03 | 0.29 ± 0.02 | 0.48 ± 0.03 | < 0.001 |
|  | 30 | 0.66 ± 0.02 | 0.55 ± 0.02 | 0.62 ± 0.02 | < 0.001 |
| Food cue | 15 | 0.32 ± 0.04 | 0.15 ± 0.02 | 0.30 ± 0.05 | 0.001 |
|  | 30 | 0.511 ± 0.03 | 0.40 ± 0.02 | 0.50 ± 0.04 | < 0.001 |

**Supplementary Table 4**

**Analysis results of individual behavior in response to food cues.** Paired sample T test was used to compare the individual behavior of sea urchins in response to food cues. Wilcox signed-rank test was used for the combinations that did not satisfy the normal distribution. The statement of the *Z* value in the table is the result of Wilcox signed-rank test.

|  |  | Speed | Displacement | *r* |
| --- | --- | --- | --- | --- |
| 1 ind/m^2^ | *t* | 3.973 | 3.270 | *Z* = -2.521 |
|  | *P* | 0.005 | 0.014 | 0.012 |
| 15 ind/m^2^ | *t* | 6.645 | 4.367 | *Z* = -6.073 |
|  | *P* | < 0.001 | < 0.001 | < 0.001 |
| 30 ind/m^2^ | *t* | 13.266 | 9.474 | *Z* = -11.061 |
|  | *P* | < 0.001 | < 0.001 | < 0.001 |

**Supplementary Table 5**

**Expansion speed *v_e_* (mm/min) of sea urchins in 15 ind/m^2^ and 30 ind/m^2^ in control and food cue periods (mean ± SEM).** Paired sample T test was used to compare the expansion speed of sea urchins in response to food cues in 15 ind/m^2^ and 30 ind/m^2^, respectively. Independent-samples T test was used to compare the expansion speed of sea urchins between 15 ind/m^2^ and 30 ind/m^2^ in control and food cue period.

|  | 15 ind/m^2^ | 30 ind/m^2^ | *t* | *P* | |
| --- | --- | --- | --- | --- | --- |
| Control | 15.44 ± 0.93 | 16.41 ± 1.56 | -0.534 | 0.602 |  |
| Food cue | 9.46 ± 0.70 | 6.71 ± 0.35 | *Z* = -2.415 | 0.015 |  |
| Paired sample T test | *t* = 4.523  *P* = 0.003 | *t* = 7.014  *P* < 0.001 |  |  |  |

**Supplementary Figure**

**Schematic graph of movement path of a sea urchin.**

**
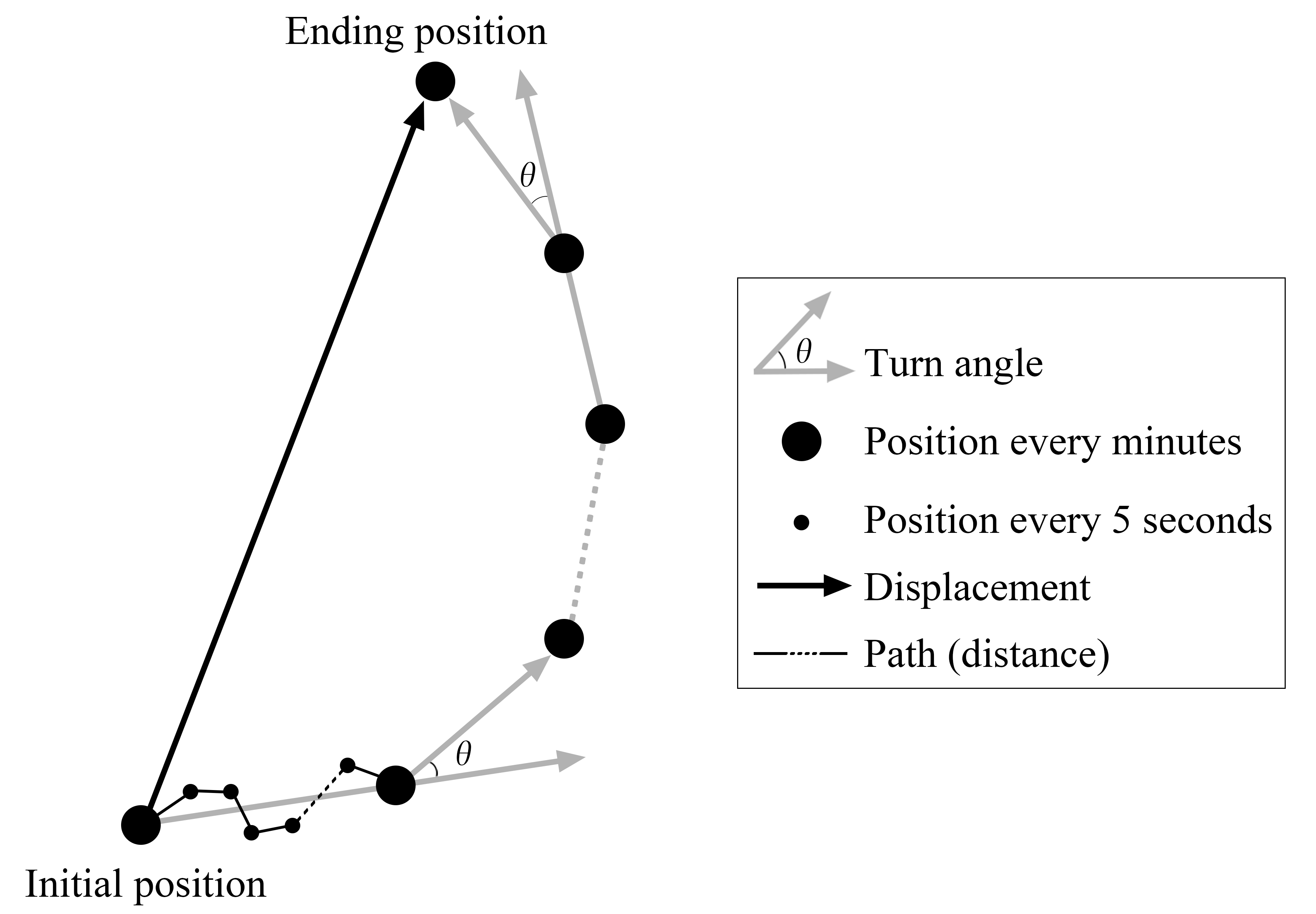
**
